# Duck MDA5 functions in innate immunity against H5N1 highly pathogenic avian influenza virus infections

**DOI:** 10.1186/1297-9716-45-66

**Published:** 2014-06-18

**Authors:** Liangmeng Wei, Jin Cui, Yafen Song, Shuo Zhang, Fei Han, Runyu Yuan, Lang Gong, Peirong Jiao, Ming Liao

**Affiliations:** 1 College of Veterinary Medicine, South China Agricultural University, Guangzhou 510642, China; 2 College of Animal Science and Veterinary Medicine, Shandong Agricultural University, Tai’an, Shandong 271018, China

## Abstract

Melanoma differentiation-associated gene 5 (MDA5) is an important intracellular receptor that recognizes long molecules of viral double-stranded RNA in innate immunity. To understand the mechanism of duck MDA5-mediated innate immunity, we cloned the MDA5 cDNA from the Muscovy duck (*Cairina moschata*). Quantitative real-time PCR analysis indicates that duck MDA5 mRNA was constitutively expressed in all sampled tissues. A significant increase of MDA5 mRNA was detected in the brain, spleen and lungs of ducks after infection with an H5N1 highly pathogenic avian influenza virus (HPAIV). We investigated the role of the predicted functional domains of MDA5. The results indicate the caspase activation and recruitment domain (CARD) of duck MDA5 had a signal transmission function through IRF-7-dependent signaling pathway. Overexpression of the CARD strongly activated the chicken IFN-β promoter and upregulated the mRNA expression of antiviral molecules (such as OAS, PKR and Mx), proinflammatory cytokines (such as IL-2, IL-6, IFN-α and IFN-γ, but not IL-1β and IL-8) and retinoic acid-inducible gene I (RIG-I)-like receptors (RLR) (RIG-I and LGP2) without exogenous stimulation. We also demonstrate the NS1 of the H5N1 HPAIV inhibited the duck MDA5-mediated signaling pathway in vitro. These results suggest that duck MDA5 is an important receptor for inducing antiviral activity in the host immune response of ducks.

## Introduction

The innate immune system (also known as the non-specific immune system) is an evolutionarily conserved system that protects the host from invading microbial pathogens and other potential threats through germline-encoded pattern recognition receptors [1]. Pattern recognition receptors are located on multiple types of innate immune cells and are capable of responding to specific pathogen associated molecular patterns exclusively present on microbes, such as viruses, bacteria, parasites and fungi. Post virus infection, some pattern recognition receptors such as toll-like receptors (TLRs; e.g., TLR-3, -7, -8 and -9), retinoic acid-inducible gene I (RIG-I)-like receptors (RLR) and nucleotide oligomerization domain-like receptors are activated, which specifically recognize various types of viral nucleotides [1,2].

The RLR family contains three members: RIG-I, melanoma differentiation-associated gene 5 (MDA5) and laboratory of genetics and physiology 2 (LGP2), which are located in the cytoplasm [3]. RLR family members harbor one central ATPase and helicase domain and one regulatory domain (RD) in the carboxy terminus. RIG-I and MDA5 also have two caspase activation and recruitment domains (CARD) at the N terminus, which is absent in LGP2 [4,5]. RIG-I recognizes short double-stranded RNA (dsRNA; < 1 kb) and uncapped 5′-triphosphate ssRNA (5′ppp-ssRNA) [6,7]. However, MDA5 senses longer dsRNA (>1 kb) and synthetic dsRNA, such as polyinosinic-polycytidylic acid (poly [I:C]) [8].

The MDA5 pathway has been well characterized in mammals. After MDA5 is activated, the signal is transmitted to interferon-β (IFN-β) promoter-stimulator-1 (IPS-1, also known as MAVS/VISA/Cardif) via a CARD-CARD interaction on the mitochondrial membrane [9-12]. This association coordinates a serine kinase-mediated cascade that activates latent transcription factors, including interferon-regulatory factor 3 (IRF-3) and nuclear factor-κB (NF-κB), culminating in the expression of IFN-β and a number of other crucial antiviral effector genes [13].

Avian influenza viruses (AIV) cause a serious and economically significant disease in domestic poultry, such as chickens, quails and pheasants. Ducks and wild birds have naturally high resistance to AIV infections [14,15], although recently there have been severe outbreaks in ducks caused by H5N1 highly pathogenic (HP) AIV [16-20]. Natural resistance to AIV in ducks has been linked molecularly to RIG-I [15]. However, whether MDA5 also has this function in ducks has not been explored until now.

Recently, a partial MDA5 sequence was deposited in GenBank for the duck (accession number: GU936632) [15]. However, full-length duck MDA5 cDNA has not been cloned and sequenced. In the present study, we cloned the full-length duck MDA5 cDNA and investigated the role of the predicted functional domains of MDA5. In addition, we investigated the MDA5-mediated signaling pathway in primary duck embryonic fibroblast (DEF) cells and examined its antiviral function. We also demonstrate that nonstructural protein 1 (NS1) of H5N1 HPAIV inhibits the MDA5-mediated signaling pathway.

## Materials and methods

### Cells, viruses, and animals

Primary DEF cells were prepared using 14-day-old Muscovy duck eggs as described previously [21]. All cells, including human embryonic kidney 293 T (China Center for Type Culture Collection, China) were maintained in Dulbecco’s modified Eagle’s medium supplemented with 10% fetal bovine serum and 1% antibiotics, and were incubated at 37 °C in 5% CO_2_.

A/Duck/Guangdong/212/2004(H5N1) virus (designated as DK212) was isolated from ducks in the Guangdong Province of China in 2004, and identified as H5N1 avian influenza A virus using hemagglutination inhibition and neuraminidase inhibition tests [20]. It was purified and propagated in the allantoic cavity of 10-day-old specific pathogen-free embryonated hen eggs. Allantoic fluid pooled from multiple eggs was clarified by centrifugation and frozen in aliquots at -70 °C. All experiments with H5N1 HPAIV were performed under animal biosafety level 3 (ABSL-3) conditions.

One-day-old healthy Muscovy ducks were purchased from a duck farm in Guangzhou and housed in isolators. Muscovy ducks were confirmed as serologically negative for avian influenza by agar gel precipitation tests and hemagglutinin inhibition assays.

### Identification of muscovy duck MDA5, PKR and OAS genes

To identify MDA5, PKR, and OAS cDNA sequences from the Muscovy duck, degenerate PCR primers were designed based on a multiple alignment of the previously reported sequences from humans, mice and chickens. PCR was performed with the degenerate primers (Table 1), using the cDNA generated from the Muscovy duck spleen. The PCR conditions comprised an initial denaturation at 94 °C for 5 min; 35 cycles of denaturation, annealing and extension at 94 °C for 30 s, 57 °C for 30 s and 72 °C for 1 min; and a final elongation step at 72 °C for 7 min. The PCR products were ligated into the pMD19-T vector (Takara, Dalian, China) and sequenced by the Shanghai Invitrogen Biotechnology Co., Ltd.

**Table 1:** Gene cloning PCR primers used in this study

**Primer name**	**Sequence of Oligonucleotide (5′ → 3′)**	**Purpose**
idMDA5-f	AGRSMTTACCARATGGAAGTKG	Gene cloning
idMDA5-r	AARTGTTCTGCACARACRCGWTC	
idPKR-f	CACCTAATTTTGATAATGCAAGAAA	Gene cloning
idPKR-r	ATAAATGTCTACTTCCTTTCCATA	
idOASL-f	TTCCTCAAGGAGCGCTGCTTC	Gene cloning
idOASL-r	GGGTCGGCGGGATCCAGGAT	
5rdM5r-1	AATGAGATTTTCAGCTGAGAATCACCAC	5′-Race
5rdM5r-2	TGATCTTTGGTAATGTAAACAG	
3rdM5f-1	GATCTCAGCCATATGAACAGTGGGTG	3′-Race
3rdM5f-2	TGATGATGATGATGAACCAGC	
dMDA5-f	ATGTCGACGGAGTGCCGAGACG	Site mutation
dMDA5-site mutation-r	AGGTGCTCTCATCAGCACGAGCTCGAC	
dMDA5-site mutation-f	GCCCGTGGTCGAGCTCGTGCTGATGAG	Site mutation
dMDA5-r	TCAGTCTTCATCACTTGAAGGACA	
pCAGGS-MDA5-f	TAA*CTCGAG*ACCATGTCGACGGAGTGCCGAGACG	MDA5 cloning
pCAGGS-MDA5-r	ATT*GCTAGC*TCACTTGTCATCGTCGTCCTTGTAGTCATCTTCATCACTTGA	
pCAGGS-MDA5ΔRD-f	TAA*CTCGAG*ACCATGTCGACGGAGTGCCGAGACG	MDA5-ΔRD cloning
pCAGGS-MDA5ΔRD-r	ATT*GCTAGC*TCACTTGTCATCGTCGTCCTTGTAGTCAGGGTTTTTCTTATATG	
pCAGGS-MDA5ΔCARD-f	TAA*CTCGAG*ACCATGACAGGAGGAAAAGAGAATAA	MDA5-ΔCARD cloning
pCAGGS-MDA5ΔCARD-r	ATT*GCTAGC*TCACTTGTCATCGTCGTCCTTGTAGTCATCTTCATCACTTGA	
pCAGGS-MDA5ΔCARD + ΔRD-f	TAA*CTCGAG*ACCATGACAGGAGGAAAAGAGAATAA	MDA5ΔCARD + ΔRD cloning
pCAGGS-MDA5ΔCARD + ΔRD-r	ATT*GCTAGC*TCACTTGTCATCGTCGTCCTTGTAGTCAGGGTTTTTCTTATATG	
pCAGGS-MDA5-CARD -f	TAA*CTCGAG*ACCATGTCGACGGAGTGCCGAGACG	MDA5-CARD cloning
pCAGGS-MDA5-CARD -r	ATT*GCTAGC*TCACTTGTCATCGTCGTCCTTGTAGTCATTTCCACTTAAATCAT	
pCAGGS-DK212-NS1-f	TAA*CTCGAG*ACCATGGATTCCAACACTGT	DK212-NS1 cloning
pCAGGS-DK212-NS1-r	ATT*GCTAGC*TCACTTGTCATCGTCGTCCTTGTAGTCAACTTTTGACTCAAT	

Note: *CGTCTC* was the recognition site of restriction enzyme *Xho I* and *GCTAGC* was the recognition site of restriction enzyme *Nhe I*. R = A/G, S = C/G, M = A/C, K = G/T, W = A/T. f = forward primer; r = reverse primer.

### Cloning and bioinformatics analysis of the full-length cDNA of MDA5

The full-length MDA5 cDNA was isolated using 5′- and 3′-SMART RACE PCR (Clontech, Mountain View, CA, USA), according to the manufacturer’s protocol, with gene-specific primers and the SMART universal primer (Table 1). The structure of deduced amino acid sequences of Muscovy duck MDA5 was analyzed using the SMART program [22]. Amino acid sequences were aligned using ClustalW2 [23] and edited with BOXSHADE [24].

### Construction of plasmids

The full-length open reading frame of Muscovy duck MDA5 was amplified from spleen cDNA by PCR with primer sets dMDA5-f and dMDA5-r. Since the position of 2397 of MDA5 has a cleavage site of *Xho I*, a silent mutation was introduced into the MDA5 gene by site-directed mutagenesis with the set of primers shown in Table 1. The mutated MDA5 products were then ligated into the pMD19-T vector and named pM-MDA5. Initially, the full-length MDA5 (residues 1-1003), the C-terminal RD deleted MDA5 (MDA5ΔRD, residues 1-876), the N-terminal CARD deleted MDA5 (MDA5ΔCARD, residues 197-1003), both C-terminal RD and N-terminal CARD deleted MDA5 (MDA5ΔCARD + ΔRD, residues 197-876), and only N-terminal CARD (MDA5-CARD, residues 1-196) were amplified from pM-MDA5 as a template using specific primer sets (Table 2): each upstream primer contained a Kozak consensus sequence, and each downstream primer contained a FLAG-tag sequence. The amplified fragments were digested with *Xho I* (Fermentas, Burlington, Ontario, Canada) and *Nhe I* (Fermentas) and ligated into *Xho I* and *Nhe I* digested pCAGGS expression vector. The constructs were termed pCAGGS-MDA5, pCAGGS-MDA5ΔRD, pCAGGS-MDA5ΔCARD, pCAGGS-MDA5ΔCARD + ΔRD, and pCAGGS-MDA5-CARD, respectively. We also generated a plasmid expressing the NS1 protein sequence of DK212 using the same method and named it pCAGGS-DK212-NS1.

**Table 2:** Quantitative real-time PCR primers used in this study

**Primer name**	**Sequence of Oligonucleotide (5′ → 3′)**	**Purpose**
qdMDA5-f	GCTACAGAAGATAGAAGTGTCA	qRT-PCR
qdMDA5-r	CAGGATCAGATCTGGTTCAG	
qdRIG-I-f	GCTACCGCCGCTACATCGAG	qRT-PCR
qdRIG-I-r	TGCCAGTCCTGTGTAACCTG	
qdLGP2-f	GTGGTGGAGCTGGAGAAGAG	qRT-PCR
qdLGP2-r	CCCTGTTCTCCTCAAAGGTG	
qdMx-f	TGCTGTCCTTCATGACTTCG	qRT-PCR
qdMx-r	GCTTTGCTGAGCCGATTAAC	
qdPKR-f	AATTCCTTGCCTTTTCATTCAA	qRT-PCR
qdPKR-r	TTTGTTTTGTGCCATATCTTGG	
qdOAS-f	TCTTCCTCAGCTGCTTCTCC	qRT-PCR
qdOAS-r	ACTTCGATGGACTCGCTGTT	
qdIL-1β-f	TCATCTTCTACCGCCTGGAC	qRT-PCR
qdIL-1β-r	GTAGGTGGCGATGTTGACCT	
qdIL-2-f	GCCAAGAGCTGACCAACTTC	qRT-PCR
qdIL-2-r	ATCGCCCACACTAAGAGCAT	
qdIL-6-f	TTCGACGAGGAGAAATGCTT	qRT-PCR
qdIL-6-r	CCTTATCGTCGTTGCCAGAT	
qdIL-8-f	AAGTTCATCCACCCTAAATC	qRT-PCR
qdIL-8-r	GCATCAGAATTGAGCTGAGC	
qdIFN-α-f	TCCTCCAACACCTCTTCGAC	qRT-PCR
qdIFN-α-r	GGGCTGTAGGTGTGGTTCTG	
qdIFN-γ-f	GCTGATGGCAATCCTGTTTT	qRT-PCR
qdIFN-γ-r	GGATTTTCAAGCCAGTCAGC	
qdGAPDH-f	ATGTTCGTGATGGGTGTGAA	qRT-PCR
qdGAPDH-r	CTGTCTTCGTGTGTGGCTGT	

Note: *qRT-PCR* = quantitative real-time polymerase chain reaction. *f* = forward primer; *r* = reverse primer.

The luciferase reporter plasmid for the chicken IFN-β (chIFN-β) promoter (pGL3-chIFNβ-Luc) has been previously described [15]. The chicken NF-κB (chNF-κB) and chicken IRF-7 (chIRF-7) binding positive regulatory domains were predicted by the TFSEARCH: Searching Transcription Factor Binding Sites [25]. The chicken pGL3-chNF-κB-Luc and pGL3-chIRF-7-Luc contain four copies of the NF-κB- (sequence: GGGAATTCTC) or IRF-7- (sequence: TTCACTTTCAATA) positive regulatory domains motif of the chicken IFN-β promoter in front of a luciferase reporter gene, respectively.

### Transient transfections and luciferase assays

DEF cells were transfected with firefly luciferase reporter plasmid, such as pGL3-chIFNβ-Luc, pGL3-chNF-κB-Luc or pGL3-chIRF-7-Luc. In addition, an internal control plasmid to normalize transfection efficiency, pTK-RL (Promega, Madison, WI, USA), encoding the Renilla luciferase protein, was transfected into the cells. The reporter gene plasmids pGL3-chIFNβ-Luc, pGL3-chNF-κB-Luc or pGL3-chIRF-7-Luc and the pTK-RL plasmids were cotransfected along with each of the pCAGGS expression plasmids into 80% confluent cells using Lipofectamine 2000 (Invitrogen, Carlsbad, CA, USA). At 24 h post-transfection, the cells were lysed and luciferase activities were determined with a dual-luciferase reporter assay system (Promega) and normalized on the basis of the Renilla luciferase activities.

### Generation of reverse genetic reassorted viruses

To establish eight-plasmid reverse genetic systems for the DK212 and DK212-ΔNS1 viruses, a bidirectional transcription vector (pDL) was used. Reassorted viruses were generated by reverse genetics as described previously [26]. The DK212 virus devoid of the NS1 gene (DK212-ΔNS1) was rescued by transfecting of 293 T cells with the plasmid pDL-NS-ΔNS1 and the seven parent DK212 plasmids. To produce a viral stock, rescued DK212 (rDK212) and rDK212-ΔNS1 were further inoculated to embryonated eggs. The rescued viruses were detected by hemagglutination assay, and RNA was extracted and analyzed by reverse transcription PCR. Each viral segment was sequenced to confirm the identity of the reassorted viruses. Viral titers were measured by 50% tissue culture infective dose (TCID_50_) on DEF cells. All experiments with H5N1 HPAIV were performed under ABSL-3 conditions.

### Quantitative real-time PCR analysis

Quantitative real-time polymerase chain reaction (qRT-PCR) was performed using the QuantiFast SYBR Green PCR kit (Qiagen, Hilden, Germany). Primers used for qRT-PCR were designed using the primer3 software [27], based on published target sequences and have been previously reported [28]. Primer pairs (Table 2) were selected based on their specificity, as determined by dissociation curves. qRT-PCR was carried out using a 7500 Fast Real-Time PCR system (Applied Biosystems, Rotkreuz, Switzerland). The relative expression ratios of target genes in the tested group versus those in the control group were calculated by the 2^-ΔΔCt^ method using the duck housekeeping gene glyceraldehyde-3-phosphate-dehydrogenase (GAPDH; AY436595) as the endogenous reference gene to normalize the level of target gene expression [29].

### Animal experiments

For tissue distribution analyses, three uninfected ducks (aged 4 weeks) were killed and tissues were collected, including the brain, crop, trachea, heart, liver, spleen, lung, kidney, muscular stomach, glandular stomach, muscle, skin, duodenum, ileum, colon, cecum, rectum and bursa.

To examine the response of duck MDA5 infected with DK212, groups of nine ducks (aged 4 weeks) were inoculated intranasally with 10^6^ EID_50_ of DK212 or mock infected with phosphate-buffered saline at a volume of 0.2 mL. At 1, 2 and 3 days post-infection (dpi), three individuals from each group were sacrificed and the brain, spleen, and lung tissues were harvested immediately for RNA extraction. The remaining ducks were observed for clinical symptoms for 14 days. All experiments were carried out in ABSL-3 facilities and were conducted under the guidance of CDC’s Institutional Animal Care and Use Committee and the Association for Assessment and Accreditation of Laboratory Animal Care International accredited facility. The ABSL-3 Committee of South China Agricultural University approved the animal experiments in this study.

### Statistical analysis

Data were expressed as means ± standard deviations. Statistical analyses were performed using the GraphPad Prism 5 software (GraphPad Software Inc., San Diego, CA, USA). A value of *P* < 0.05 was considered significant.

## Results

### Muscovy duck MDA5 cDNA and gene structure

**Figure 1:**
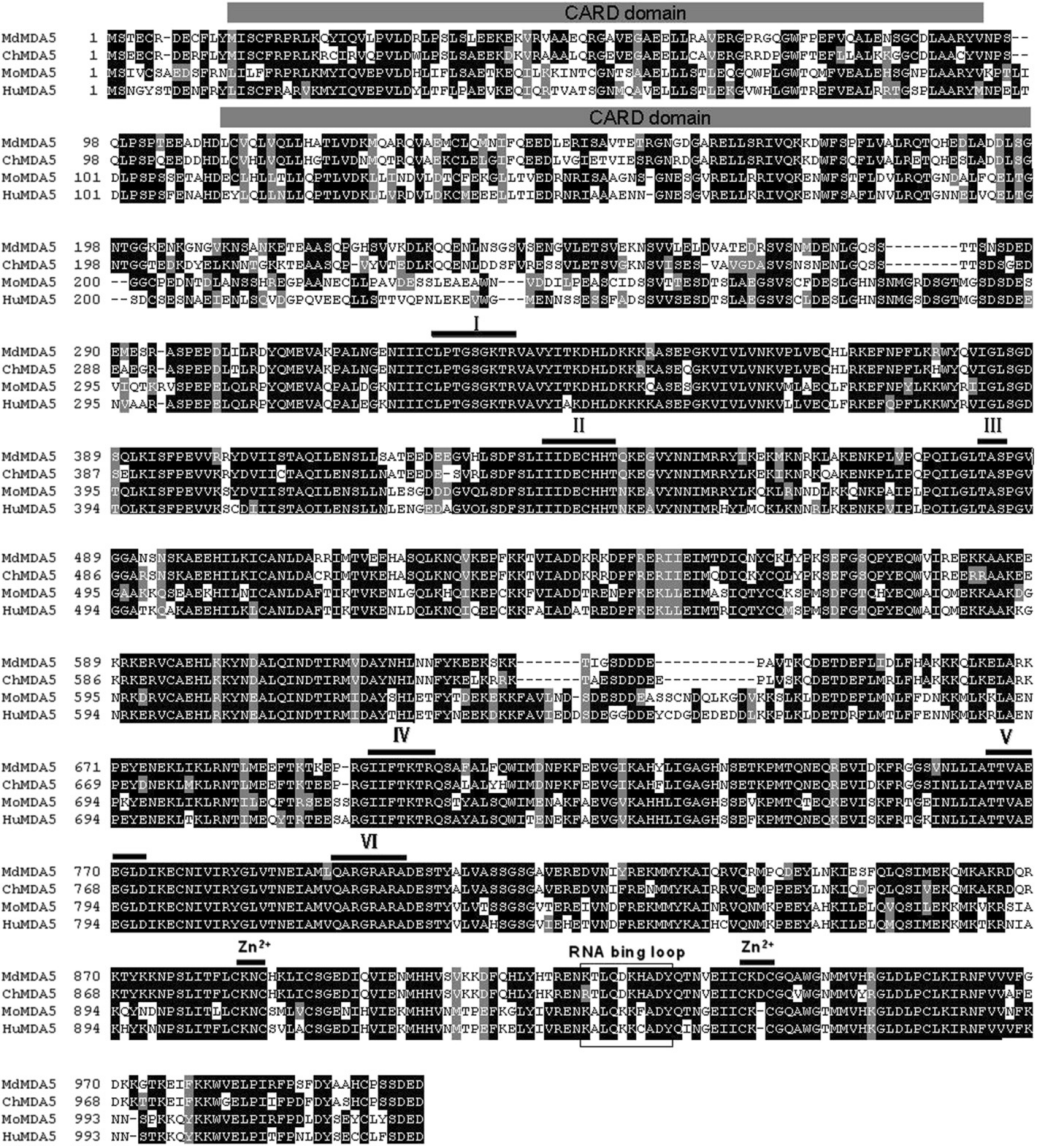
**Amino acid sequence alignment of Muscovy duck MDA5 protein with other MDA5 proteins.** Accession numbers: human (accession numbers; NP_071451), mouse (NP_082111) and chicken (NP_001180567). The predicted motifs for CARD are indicated with gray bars on the alignment. The predicted motifs for DExD/H box RNA helicase domains are indicated by numbers (I-VI) and lines. Two predicted Zn^2+^-binding motifs and an RNA binding loop are indicated with black lines and a box. MDA5 sequences are shown for Muscovy duck (Md), human (Hu), mouse (Mo) and chicken (Ch).

Homologous cloning and the RACE technique were used to obtain the full-length MDA5 cDNA sequence from a spleen cDNA library of the Muscovy duck. The open reading frame of MDA5 comprised 3012 bp (GenBank accession number: KF709945) encoding a protein of 1003 aa. As shown in Figure 1, the domain architecture of Muscovy duck MDA5 contains two CARD motifs in the N-terminal region (located at positions 13-94 and 110-197), a type III restriction enzyme domain (position 300-488), a helicase conserved C-terminal domain (position 719-802) and an RIG-I C-terminal regulatory domain (position 877-1000) containing two Zn^2+^-binding regions (positions 883-886 and 938-941) and one RNA-binding loop (positions 921-930). The RNA helicase region is highly similar to the RNA helicase regions in other vertebrate MDA5 (Figure 1), especially functional motifs I-VI, which are required for ATP hydrolysis and RNA binding in mammalian RLR [30]. At the amino acid sequence level, the full-length Muscovy duck MDA5 is highly similar to the MDA5 of other vertebrates: 86.1% identity with chickens, 63.5% with humans, and 63.2% with mice (Figure 1).

### Tissue distribution of Muscovy duck MDA5 expression

To investigate the expression of MDA5 mRNA in Muscovy duck normal tissues, a qRT-PCR analysis was performed. The Muscovy duck MDA5 mRNA showed widespread expression in the tested tissues. It was strongly expressed in the trachea, ileum, duodenum, crop, rectum and colon, and weakly expressed in the brain, heart, skin and muscle (Table 3).

**Table 3:** **Quantitative analysis of tissue distribution of MDA5 transcripts in healthy Muscovy ducks**^**1**^

**Tissue**	**MDA5 gene relative expression level (mean ± SD)**	**Tissue**	**MDA5 gene relative expression level (mean ± SD)**
Brain	0.62 ± 0.09^a^	Glandular stomach	8.16 ± 0.71^a^
Crop	19.61 ± 1.57^a^	Muscle	0.02 ± 0.004^a^
Trachea	373.68 ± 13.83^a^	Skin	0.07 ± 0.006^a^
Heart	0.13 ± 0.01^a^	Duodenum	22.26 ± 1.89^a^
Liver	1.62 ± 0.19^a^	Ileum	23.76 ± 0.66^a^
Spleen	2.68 ± 0.17^a^	Colon	13.88 ± 0.84^a^
Lung	1.45 ± 0.13^a^	Caecum	6.98 ± 0.58^a^
Kidney	2.02 ± 0.17^a^	Rectum	15.02 ± 0.86^a^
Muscular stomach	0.41 ± 0.05^a^	Bursa	1.00 ± 0.11

^1^Each result represents the level of target gene mRNA relative to those in the bursa, expressed as the mean ± SD (standard deviation) of triplicate from qRT-PCR assays. ^a^indicates there was a difference (*P* < 0.05) between control and test tissues.

### Expression of MDA5 in muscovy ducks infected with DK212

**Figure 2:**
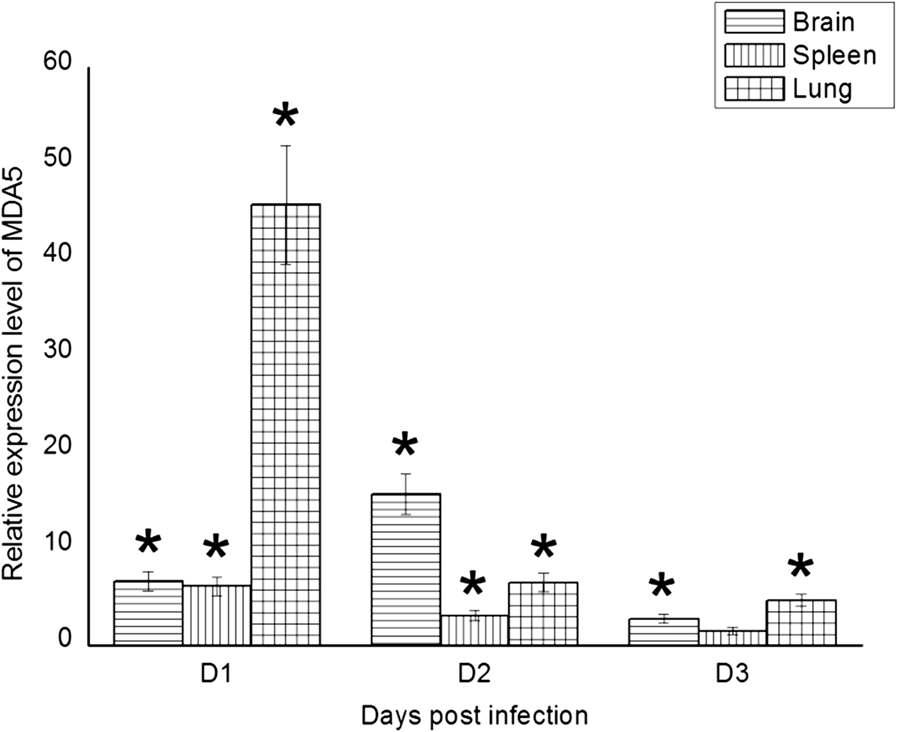
**The relative mRNA expression patterns of Muscovy duck MDA5 in the brain, spleen and lung by qRT-PCR.** The controls were inoculated with PBS; the experimental ducks were infected with DK/212. Each bar represents the level of the target gene mRNA relative to those in the control group. *The difference (*P* < 0.05) between the experimental and control group. Error bars indicate standard deviations.

To confirm whether MDA5 was involved in the host antiviral response to H5N1 HPAIV infection in ducks, we measured the mRNA expression of MDA5 in the brain, spleen and lungs of Muscovy ducks following infection with DK212. In the brain, the expression of MDA5 was significantly upregulated at 1 dpi (6.58-fold, *P* < 0.05), reached a peak at 2 dpi (15.67-fold, *P* < 0.05), and then decreased at 3 dpi (2.69-fold, *P* < 0.05) (Figure 2). In the spleen, the expression of MDA5 was significantly upregulated at 1 dpi (6.58-fold, *P* < 0.05), decreased a little at 2 dpi (3.01-fold, *P* < 0.05), and recovered to almost a normal level at 3 dpi (1.42-fold) (Figure 2). In the lungs, the expression of MDA5 was significantly upregulated at 1 dpi (45.79-fold, *P* < 0.05), and decreased at 2 and 3 dpi (6.46-fold and 4.62-fold, respectively. *P* < 0.05) (Figure 2).

### Function of the domain of muscovy duck MDA5 in type I IFN induction

**Figure 3:**
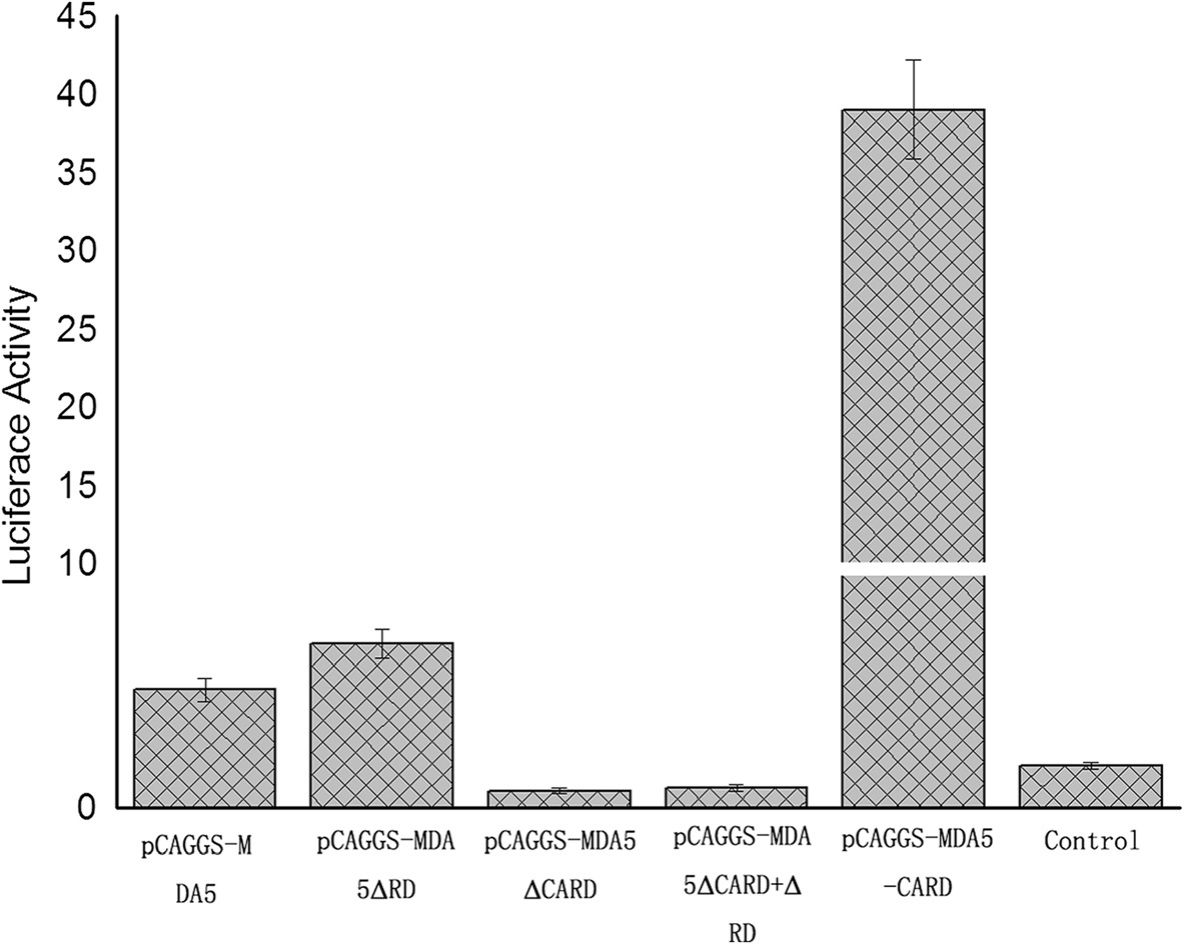
**Effects of different functional domains of Muscovy duck MDA5 on the chicken IFN-β promoter.** The pCAGGS-MDA5, pCAGGS-MDA5ΔRD, pCAGGS-MDA5ΔCARD, pCAGGS-MDA5ΔCARD + ΔRD, pCAGGS-MDA5-CARD or the empty plasmid was cotransfected with pGL3-chIFNβ-Luc and pTK-RL plasmid in DEF cells. Dual-luciferase assays were performed after 36 h.

As shown in Figure 3, the overexpression of the CARD of Muscovy duck MDA5 (pCAGGS-MDA5-CARD) significantly induced the chIFN-β promoter in DEF cells; however, the overexpression of the Muscovy duck MDA5 lacking the CARD (pCAGGS-MDA5ΔCARD or pCAGGS-MDA5ΔCARD + ΔRD) failed to induce the chIFN-β promoter. In addition, the overexpression of the Muscovy duck MDA5 lacking the predicted C-terminal RD (pCAGGS-MDA5ΔRD) induced the expression of the chIFN-β promoter more strongly than the full-length Muscovy duck MDA5 (pCAGGS-MDA5) (Figure 3). Taken together, these data strongly indicate a critical requirement of the CARD in Muscovy duck MDA5 for inducing type I IFN and that the C-terminal RD in Muscovy duck MDA5 has self-repression ability.

### Muscovy duck MDA5 signaling through the IRF-7 pathway

**Figure 4:**
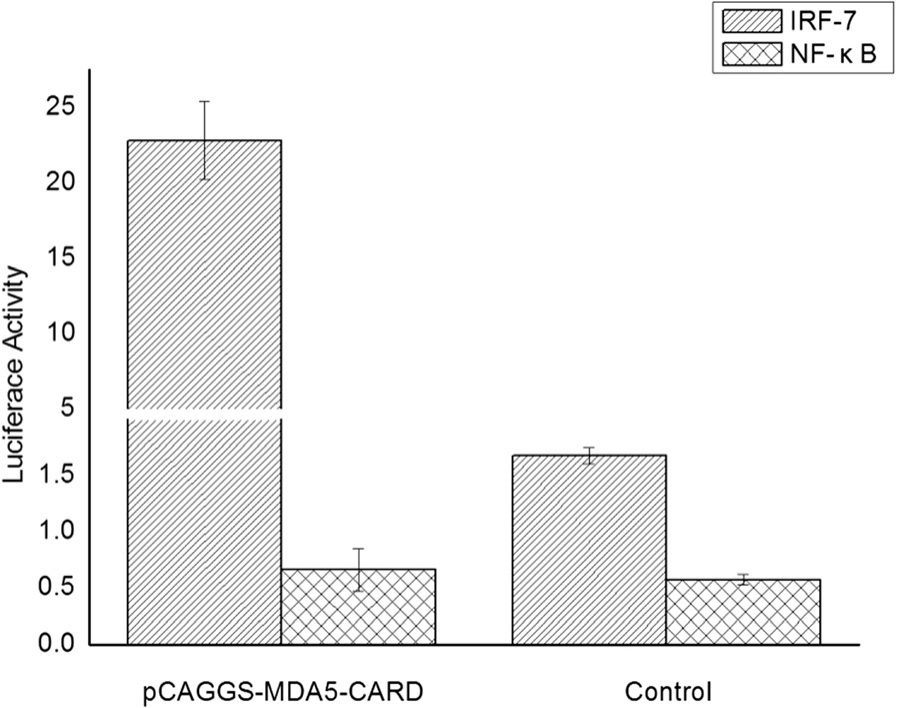
**Effects of the CARD of Muscovy duck MDA5 on the chicken IRF-7 and NF-κB promoters.** The DEF cells were cotransfected with pGL3-chIRF-7-Luc (or pGL3-chNF-κB-Luc) and the pTK-RL plasmid and with pCAGGS-MDA5-CARD or with the empty plasmid. Dual-luciferase assays were performed after 36 h.

To evaluate whether MDA5 was involved in NF-κB and/or IRF-7 signaling pathways, the DEF cells were cotransfected with pGL3-chIRF-7-Luc (or pGL3-chNF-κB-Luc) and the pTK-RL plasmid and with pCAGGS-MDA5-CARD or with the empty plasmid. The results indicate that overexpression of the CARD of Muscovy duck MDA5 significantly activated the IRF-7-dependent signaling pathway in DEF cells (Figure 4). However, the NF-κB-dependent signaling pathway did not significantly change after overexpression of the CARD domain of Muscovy duck MDA5 (Figure 4). These results suggest that Muscovy duck MDA5 is involved in signaling mainly through the IRF-7 pathway.

### Antiviral molecules and proinflammatory cytokine responses induced by CARD overexpression

**Figure 5:**
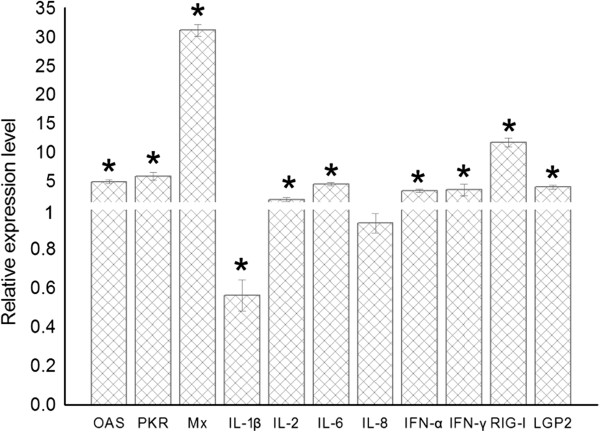
**Primary DEF cells respond to overexpression of the CARD of Muscovy duck MDA5.** The relative mRNA expression level of antiviral molecules (OAS, PKR and Mx), proinflammatory cytokines (IL-1β, IL-2, IL-6, IL-8, IFN-α and IFN-γ) and RLR (RIG-I and LGP2) post overexpression of the CARD of Muscovy duck MDA5 by qRT-PCR. The DEF cells were transfected with either the pCAGGS-MDA5-CARD plasmid or the empty plasmid. After 36 h quantitative real-time PCR tests were performed. *The difference (*P* < 0.05) between the experimental and control group. Error bars indicate standard deviations.

To understand the downstream response of the RLR pathway induced by MDA5, the gene mRNA expressions of antiviral molecules (such as OAS, PKR and Mx) and proinflammatory cytokines (such as IL-1β, IL-2, IL-6, IL-8, IFN-α and IFN-γ) were examined in DEF cells. The expression of all examined antiviral molecule genes were significantly induced by overexpression of the CARD of the Muscovy duck. The Mx gene showed the strongest expression (Figure 5). The expression patterns of the examined proinflammatory cytokines changed in response to overexpression of the CARD of the Muscovy duck. The expressions of IL-2, IL-6, IFN-α and IFN-γ were significantly induced by CARD overexpression (Figure 5); however, the expressions of IL-1β and IL-8 did not significantly change compared with their expression in cells transformed with empty plasmids (Figure 5). These data indicate that the CARD of Muscovy duck MDA5 was a strong inducer of antiviral molecules (such as OAS, PKR and Mx) and proinflammatory cytokines (such as IL-2, IL-6, IFN-α and IFN-γ).

### Overexpression of CARD upregulates the expression of RIG-I and LGP2

Regulation of the expressions of other RLR genes by overexpression of the CARD of the Muscovy duck was studied in vitro using DEF cells. Upon overexpression of the CARD of the Muscovy duck, the levels of RIG-I and LGP2 mRNA transcripts increased compared with their levels in cells transformed with the empty plasmid (Figure 5). These data indicate that activation of the Muscovy duck MDA5 pathway upregulated the mRNA expression of RIG-I and LGP2.

### Suppression of viral yield by CARD overexpression

**Figure 6:**
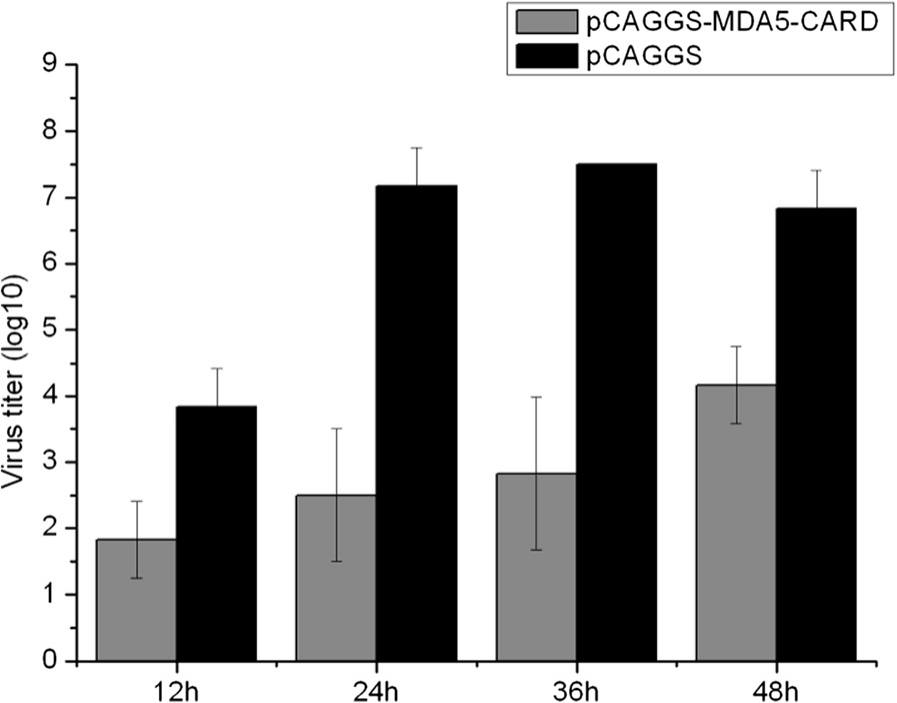
**Primary DEF cells respond to infection of DK212.** The DEF cells were transfected with either the pCAGGS-MDA5-CARD plasmid or the empty plasmid. After 16 h, the cells were infected at a dose of 5 TCID_50_ by DK212. Supernatants were collected at several time-points and analyzed for their TCID_50_ titers. Virus titers are expressed as means ± standard deviation in log_10_ TCID_50_/mL. For statistical purposes, a value of 1.5 was assigned if the virus was not detected from the undiluted sample.

To evaluate the antiviral activity of Muscovy duck MDA5, DEF cells were transfected with either the pCAGGS-MDA5-CARD plasmid or the empty plasmid, and then inoculated with DK212. As shown in Figure 6, the virus titers of DEF cells transfected with pCAGGS-MDA5-CARD vector were lower than those in cells transfected with the empty vector at all the tested time points. These data suggest that activation of the MDA5 pathway inhibited the replication of DK212 in DEF cells.

### The NS1 of DK212 inhibits the muscovy duck MDA5 signaling pathway

**Figure 7:**
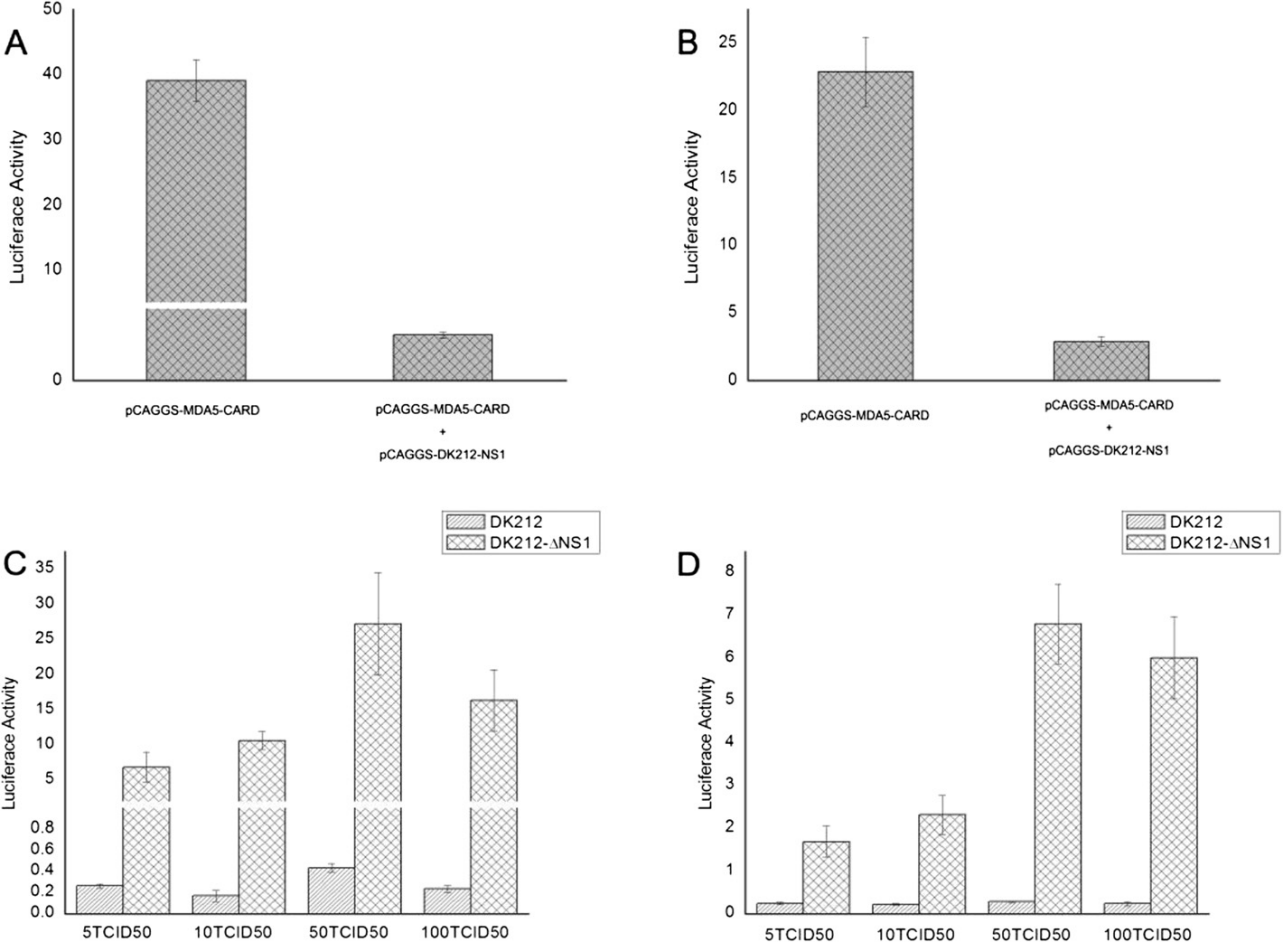
**H5N1 HPAIV NS1 interferes with chIFN-β and chIRF-7 induction.** The DEF cells were cotransfected with **(A)** pGL3-chIFNβ-Luc or **(B)** pGL3-chIRF-7-Luc and the pTK-RL plasmid and with pCAGGS-MDA5-CARD and with pCAGGS-DK212-NS1 or with the empty plasmid. Dual-luciferase assays were performed after 36 h. The DEF cells were cotransfected with **(C)** pGL3-chIFNβ-Luc or **(D)** pGL3-chIRF-7-Luc and the pTK-RL plasmid. After 12 h, the cells were infected with DK212 or rDK212-ΔNS1 at different doses. Dual-luciferase assays were performed after 24 h.

**Figure 8:**
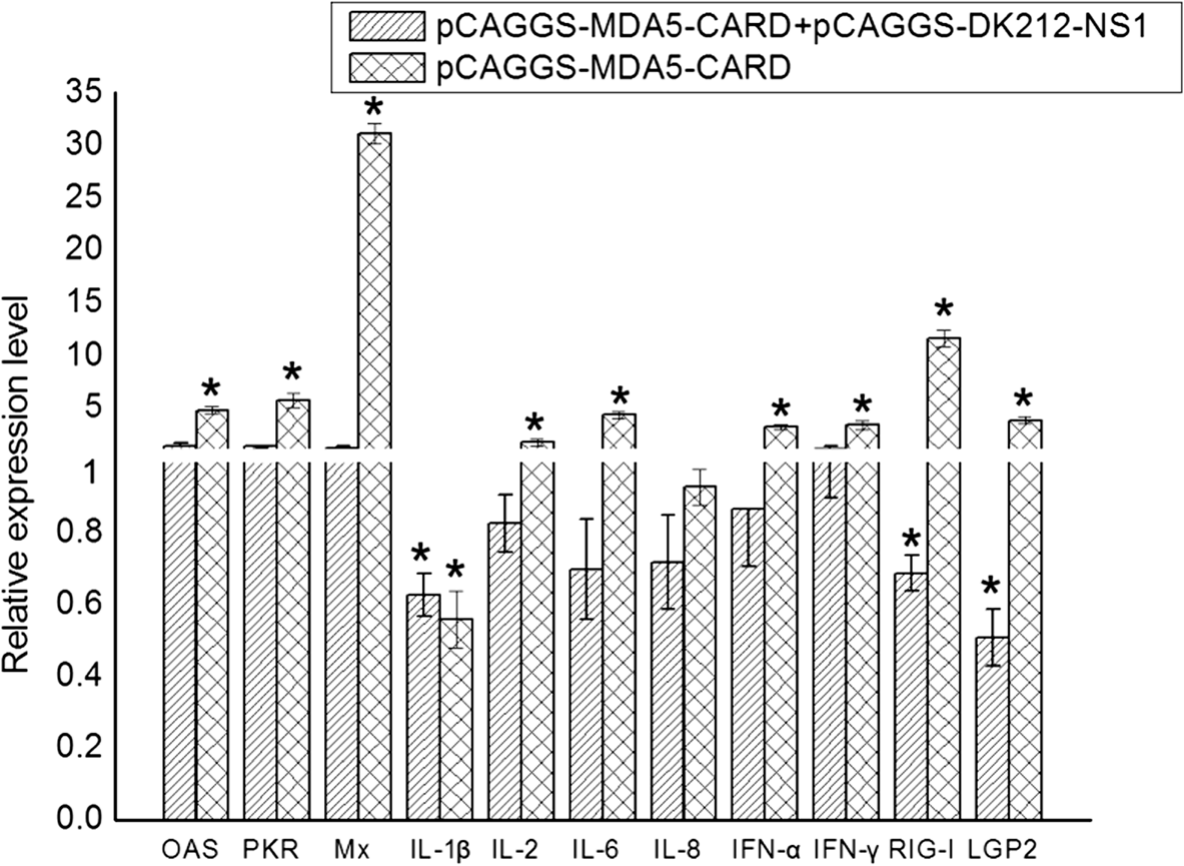
**H5N1 HPAIV NS1 interferes with antiviral molecules, proinflammatory cytokines and RLR induction.** The DEF cells were cotransfected with pCAGGS-MDA5-CARD and with pCAGGS-DK212-NS1 or with the empty plasmid. The control group was only transfected with pCAGGS. After 24 h quantitative real-time PCR tests of antiviral molecules (OAS, PKR and Mx), proinflammatory cytokines (IL-1β, IL-2, IL-6, IL-8, IFN-α and IFN-γ) and RLR (RIG-I and LGP2) were performed. *The difference (*P* < 0.05) between the experimental and control group. Error bars indicate standard deviations.

**Figure 9:**
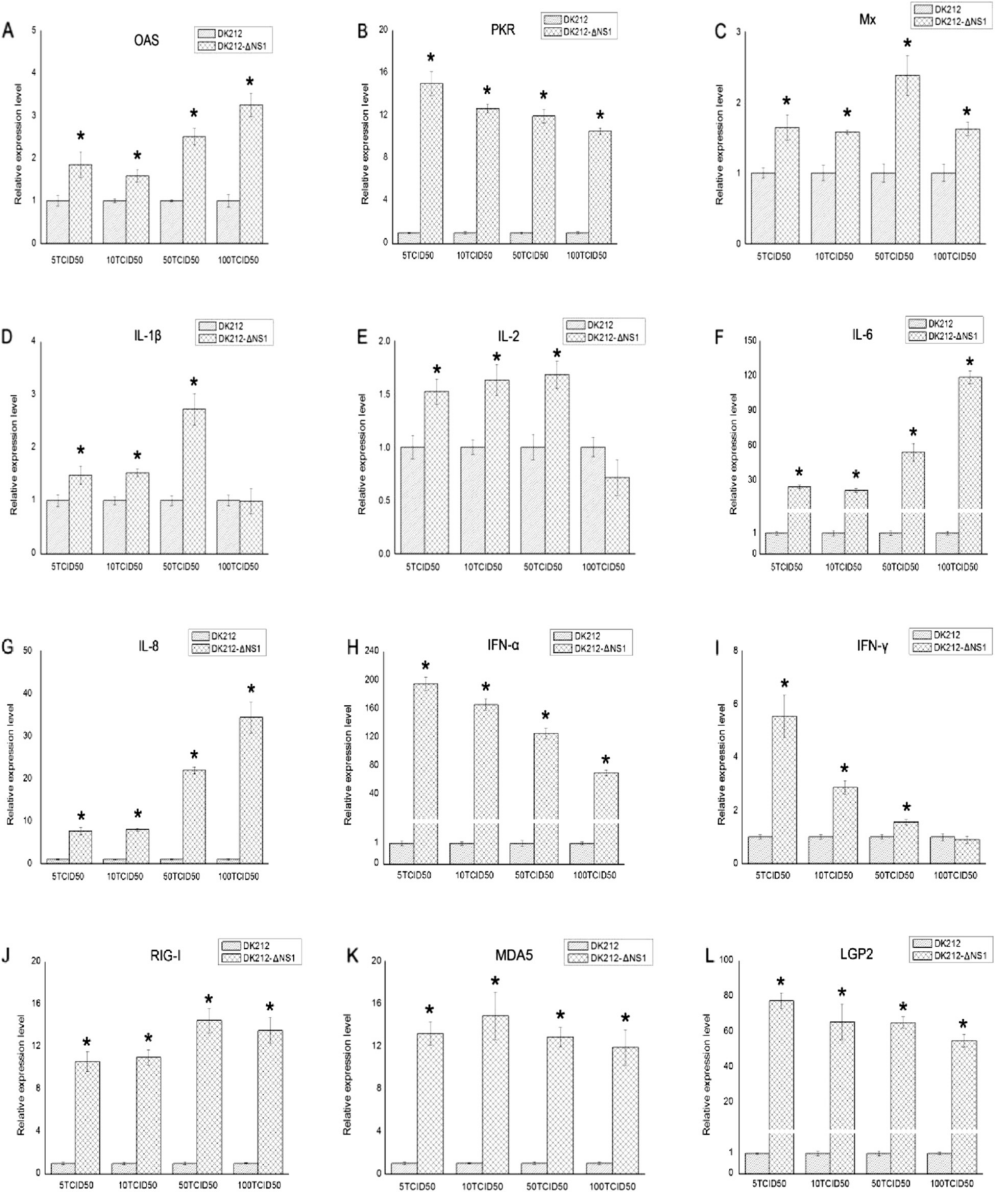
**H5N1 HPAIV NS1 interferes with (A-C) antiviral molecules, (D-I) proinflammatory cytokines and (J-L) RLR induction.** DEF cells were infected with DK212 or rDK212-ΔNS1 at different doses. After 24 h quantitative real-time PCR tests were performed. *The difference (*P* < 0.05) between the experimental and control group. Error bars indicate standard deviations.

In mammals, influenza NS1 is described as a nonessential virulence factor, able to inhibit the innate antiviral immune response of infected cells. In ducks, the interaction between the NS1 of H5N1 HPAIV and the innate immune response remains to be evaluated. In this study, overexpression of the NS1 of DK212 prevented the Muscovy duck MDA5-mediated induction of the chIFN-β promoter in DEF cells (Figure 7A). The Muscovy duck MDA5-mediated induction of the IRF-7 promoter was also prevented by overexpression of NS1 (Figure 7B). In addition, the Muscovy duck MDA5-mediated antiviral molecule and proinflammatory cytokine (Figure 8) responses were completely prevented by the overexpression of NS1. Furthermore, the upregulation of RIG-I and LGP2 were also prevented by overexpression of NS1 (Figure 8).To further illustrate the importance of the NS1 as an antagonist of the host innate immune response in ducks, we used DK212 as a backbone to generate an NS1 deleted virus, named rDK212-ΔNS1, by reverse genetics. The mutant virus was a very potent chIFN-β promoter inducer compared with the wild-type virus when infecting DEF cells (Figure 7C). In addition, the IRF-7 pathway was activated post infection with rDK212-ΔNS1, but not with DK212 (Figure 7D). Furthermore, rDK212-ΔNS1 was a potent inducer of antiviral molecules (such as OAS, PKR and Mx) (Figure 9A-C), proinflammatory cytokines (IL-1β, IL-2, IL-6, IL-8, IFN-α and IFN-γ) (Figure 9D-I) and RLR (such as RIG-I, MDA5 and LGP2) (Figure 9J-L) compared with the wild-type virus when infecting DEF cells.

Taken together, these data show that the NS1 functions to prevent MDA5-mediated innate immunity in Muscovy duck cells and underlines the need for a functional NS1 for full virulence.

## Discussion

Here, we successfully cloned the 3012 bp MDA5 cDNA from the Muscovy duck. The amino acid sequence alignment shows that duck MDA5 is highly homologous to the antiviral molecule chicken MDA5 (86.1%), suggesting that duck MDA5 may have a similar function. Based on the predicted amino acid sequence, bioinformatic analysis was performed. Like other MDA5 [4,31,32], duck MDA5 contained three main structural domains: two CARD motifs at the N terminus, one DExD/H-box RNA helicase domain and an RD at the C-terminus.

The functional profile of the predicted domains of Muscovy duck MDA5 was examined in this study. Overexpression of the CARD of duck MDA5 significantly induced the chIFN-β promoter, which is consistent with the function of MDA5 in mammalian systems [33,34]. However, overexpression of the Muscovy duck MDA5 lacking the CARD failed to induce the chIFN-β promoter (Figure 3). These results demonstrate the CARD of Muscovy duck MDA5 has a similar function as its counterpart in mammals, which is involved in immune signaling [4,5,30]. In addition, overexpression of the Muscovy duck MDA5 lacking the predicted C-terminal RD showed a stronger ability to induce the chIFN-β promoter than expression of the full-length duck MDA5 (Figure 3). This result demonstrates that the C-terminal RD of Muscovy duck MDA5 has the self-repression ability. However, the C-terminal RD of human MDA5 neither stimulates nor inhibits IFN-β promoter expression [7].The overexpression of the CARD of Muscovy duck MDA5 significantly activated the IRF-7-dependent signaling pathway (Figure 4). In addition, the expressions of several antiviral molecule genes, such as Mx, PKR and OAS, were significantly induced by overexpression of the CARD of Muscovy duck MDA5. The expression of the Mx gene was induced more strongly than others (Figure 5). In addition, the expressions of IL-2, IL-6, IFN-α and IFN-γ were significantly induced by overexpression of the CARD of Muscovy duck MDA5 (Figure 5). Therefore, Muscovy duck MDA5 activates signaling pathways that mediate both antiviral and proinflammatory responses.

Overexpression of the CARD of Muscovy duck MDA5 also increased the expressions of RIG-I and LGP2 transcripts (Figure 5). It was reported that IFN-α and IFN-β treatment of a human lung adenocarcinoma epithelial cell line and human umbilical vein endothelial cells resulted in activation of MDA5, RIG-I, and TLR3 mRNA expression [35]. Previous research also showed that the MDA5 and RIG-I genes are upregulated by IFN-α [33,36,37]. Thus, the upregulation of RIG-I and LGP2 genes induced by overexpression of CARD may be caused by the upregulation of IFN-α and IFN-β.

Muscovy duck MDA5 mRNA was constitutively expressed in all tested tissues of healthy ducks (Table 3), and was induced post infection with H5N1 HPAIV (Figure 2). These findings indicate that duck MDA5 might be an important receptor for recognizing H5N1 HPAIV in the antiviral innate immune response in Muscovy ducks. The results of in vitro antiviral assays of duck MDA5 show that virus titers of DK212 in the supernatant of CARD-overexpressing DEF cells were significantly lower than in the control (Figure 6). These results suggest that activation of the MDA5 pathway inhibited the replication of H5N1 HPAIV in vitro. It was reported that chicken cells, including DF-1 fibroblasts and HD-11 macrophage-like cells, used MDA5 to sense AIV [38]. Chickens lack RIG-I, but can regulate the production of IFN-β through the MDA5 pathway [39]. Our results suggest that Muscovy duck MDA5 has a similar function to chicken MDA5; i.e., it is involved in the host antiviral innate immunity.

In mammals, influenza NS1 is described as a multifunctional protein with regulatory activities, able to inhibit the innate antiviral immune response of infected cells [40]. In ducks, the interaction between NS1 and the innate immune response remains to be evaluated [41]. Here, we showed that in the duck system, NS1 interferes with the duck MDA5-mediated pathway. The plasmid-expressed NS1 of DK212 suppressed duck MDA5-mediated chIFN-β promoter activation. This was similar to the results reported in chicken cells, where plasmid-expressed NS1 suppressed chicken MDA5-mediated chIFN-β promoter activation [38]. In addition, plasmid-expressed NS1 of DK212 also suppressed the duck MDA5-mediated proinflammatory and antiviral signaling pathways. These results were similar to those reported in mammals [40].

In previous works, several groups have shown that viruses expressing a truncated NS1 elicited a major type I IFN response in infected cells compared with their wild-type counterparts [42-47]. Using reverse genetics, we successfully created a mutant H5N1 AIV with a deleted NS1 protein (rDK212-ΔNS1). The mutant virus, but not the wild-type virus, was a strong IFN inducer in DEF cells (Figure 7C). A similar result was reported in DEF cells in which a mutant H7N1 AIV expressed a truncated NS1 protein that induced high titers of type I IFN [41]. In addition, the mutant virus (rDK212-ΔNS1) was an effective inducer of both antiviral molecules and proinflammatory cytokines. These results once again demonstrated that NS1 of DK212 inhibited the duck MDA5-mediated signaling pathway.

In summary, we cloned the Muscovy duck MDA5 cDNA and demonstrate that the CARD of Muscovy duck MDA5 has a similar function as its mammalian counterpart, which is involved in immune signaling. In addition, overexpression of the CARD of Muscovy duck MDA5 activated the IRF-7-dependent signaling pathway and mediated both antiviral and proinflammatory responses in DEF cells. Importantly, overexpression of the CARD of the Muscovy duck MDA5 led to induction of the chIFN-β promoter and inhibition of the replication of H5N1 HPAIV in vitro. We also demonstrate that NS1 of H5N1 HPAIV inhibits the duck MDA5-mediated signaling pathway. This molecular cloning and functional characterization of the Muscovy duck MDA5 increases our understanding of the host immune response of ducks infected with H5N1 HPAIV.

## Competing interests

The authors declare that they have no competing interests.

## Authors’ contributions

LMW and PRJ participated in the design of the study, performed the experiments, collected and analyzed data, and drafted the manuscript. JC and YFS cloned and sequenced duck MDA5 cDNA. SZ and FH helped with the animal experiment. RYY and LG participated in preliminary data acquisition and performed the statistical analysis. ML conceived the study and participated in its design and coordination. All authors read and approved the final manuscript.
